# VEGFR1 Activity Modulates Myeloid Cell Infiltration in Growing Lung Metastases but Is Not Required for Spontaneous Metastasis Formation

**DOI:** 10.1371/journal.pone.0006525

**Published:** 2009-09-18

**Authors:** Michelle R. Dawson, Dan G. Duda, Sung-Suk Chae, Dai Fukumura, Rakesh K. Jain

**Affiliations:** Steele Laboratory for Tumor Biology, Massachusetts General Hospital, Harvard Medical School, Boston, Massachusetts, United States of America; Bauer Research Foundation, United States of America

## Abstract

The role of vascular endothelial growth factor receptor 1 (VEGFR1/Flt1) in tumor metastasis remains incompletely characterized. Recent reports suggested that blocking VEGFR1 activity or the interaction with its ligands (VEGF and PlGF) has anti-tumor effects. Moreover, several studies showed that VEGFR1 mediates tumor progression to distant metastasis. All these effects may be exerted indirectly by recruitment of bone marrow-derived cells (BMDCs), such as myeloid cells. We investigated the role of VEGFR1 activity in BMDCs during the pre-metastatic phase, i.e., prior to metastatic nodule formation in mice after surgical removal of the primary tumor. Using pharmacologic blockade or genetic deletion of the tyrosine kinase domain of VEGFR1, we demonstrate that VEGFR1 activity is not required for the infiltration of *de novo* myeloid BMDCs in the pre-metastatic lungs in two tumor models and in two mouse models. Moreover, in line with emerging clinical observations, we show that blockade of VEGFR1 activity neither prevents nor changes the rate of spontaneous metastasis formation after primary tumor removal. Prevention of metastasis will require further identification and exploration of cellular and molecular pathways that mediate the priming of the metastatic soil.

## Introduction

VEGF, and more recently, PlGF, have been shown to play important roles in tumor angiogenesis in preclinical studies. Moreover, VEGF is a clinically validated target for antiangiogeneic therapy for cancer, and agents that block PlGF or the tyrosine kinase activity of their cognate receptor VEGFR1 are currently approved for cancer treatment or in clinical trials (sunitinib, sorafenib, cediranib, axitinib, pazopanib, BIBF1120, etc.) [1], [2], [3]. In addition to the roles of VEGFR1 activation in tumor endothelial cells, it has been hypothesized that VEGFR1 activation mediates the mobilization of bone marrow-derived cells (BMDCs) into blood circulation [4]. Other studies have shown that BMDCs are recruited to certain tumors and facilitate tumor progression [5], [6]. A recent study demonstrated that PlGF, a ligand for VEGFR1 as well as Neuropilins 1 and 2 (NRP1/2), significantly modulated the recruitment of macrophages, tumor growth and local invasion [7]. On the other hand, blockade of VEGFR1 did not affect BMDC accumulation or growth of pancreatic endocrine tumors [8]. Moreover, VEGFR1 blockade may differentially affect the recruitment of various BMDC populations in tumors. For example, cediranib, an agent that potently inhibits VEGFR1 activity, transiently reduced macrophage infiltration but increased the total number of myeloid (CD11b^+^) cells and did not delay the growth rate of brain tumors [9]. Thus, the benefit of targeting VEGFR1 activity remains unclear, and is likely to be highly tumor–, BMDC type– and context-dependent.

In addition to effects at the primary tumor site, blockade of VEGFR1 has been proposed as an anti-metastasis approach. Previous studies in *flt1*-tk deficient mice have shown that MMP-9 is induced by VEGFR1 signaling in lung cells and facilitates metastatic tumor growth in experimental metastasis models (i.e., after intravenous infusion of cancer cells). Moreover, it has been recently reported that BMDCs – systemically mobilized in response to primary tumor growth – home to the lungs and form “pre-metastatic niches” in lungs even prior to the arrival of metastatic cancer cells [10]. A critical mediator of myeloid (CD11b^+^) BMDC recruitment to the “pre-metastatic niche” was shown to be hypoxia-induced lysyl oxidase [11]. Hypoxia is known to induce VEGF, PlGF, and their cognate receptor VEGFR1 [1], [12]. In a recent study, blockade of VEGFR1 with a specific antibody, MF1 (ImClone Systems, Inc.) has been shown to inhibit lung infiltration by BMDCs, subsequent “pre-metastatic niche” formation and metastatic tumor growth [10].

Spontaneous metastasis formation can be induced in preclinical models by surgically removing metastatic primary tumors [13], [14], [15], [16]. This model, relevant for resectable human cancers, has been extensively used to study the process of metastasis, including the role of BMDCs [13], [14]. In this model, we found that blockade of VEGFR1 activity did not affect the rate of spontaneous metastasis formation after primary tumor removal [17]. Here, we show that myeloid (CD11b^+^) BMDCs (e.g., pulmonary alveolar macrophages), are present in normal lungs and that VEGFR1 blockade does not modulate their infiltration in the presence of primary tumors prior to metastasis formation. On the other hand, we show that BMDCs may be affected by VEGFR1 activity blockade at later time-points, i.e., during metastatic tumor growth. Thus, while VEGFR1 activity is not required for the formation of metastatic tumor nodules, its blockade may differentially modulate the BMDC infiltration and growth of primary tumors and metastatic nodules.

## Materials and Methods

### Mice

C57BL and Actb-GFP/C57BL mice (constitutively expressing GFP) were obtained from the Jackson Laboratory (Bar Harbor, Maine). *Flt-1* ^TK–/–^/C57BL mice were backcrossed to 99.9% C57BL strain background (N10 equivalent) from *flt-1* ^TK–/–^ mice [18], [19], kindly provided by Dr. M. Shibuya, University of Tokyo, Japan. Strain background was verified by the Jackson Laboratory's Speed Congenic Development Service (The Jackson Laboratory, Bar Harbor, Maine). All mice were bred and maintained in sterile barrier animal facilities. All *flt-1* ^TK–/–^/C57BL mice were used for experiments after genotyping to confirm the deletion of the intracellular domain of VEGFR1. All animal experiments were performed after obtaining approval from the Subcommittee for Research and Animal Care of the Massachusetts General Hospital.

### Bone Marrow Transplantation

**Figure 1:**
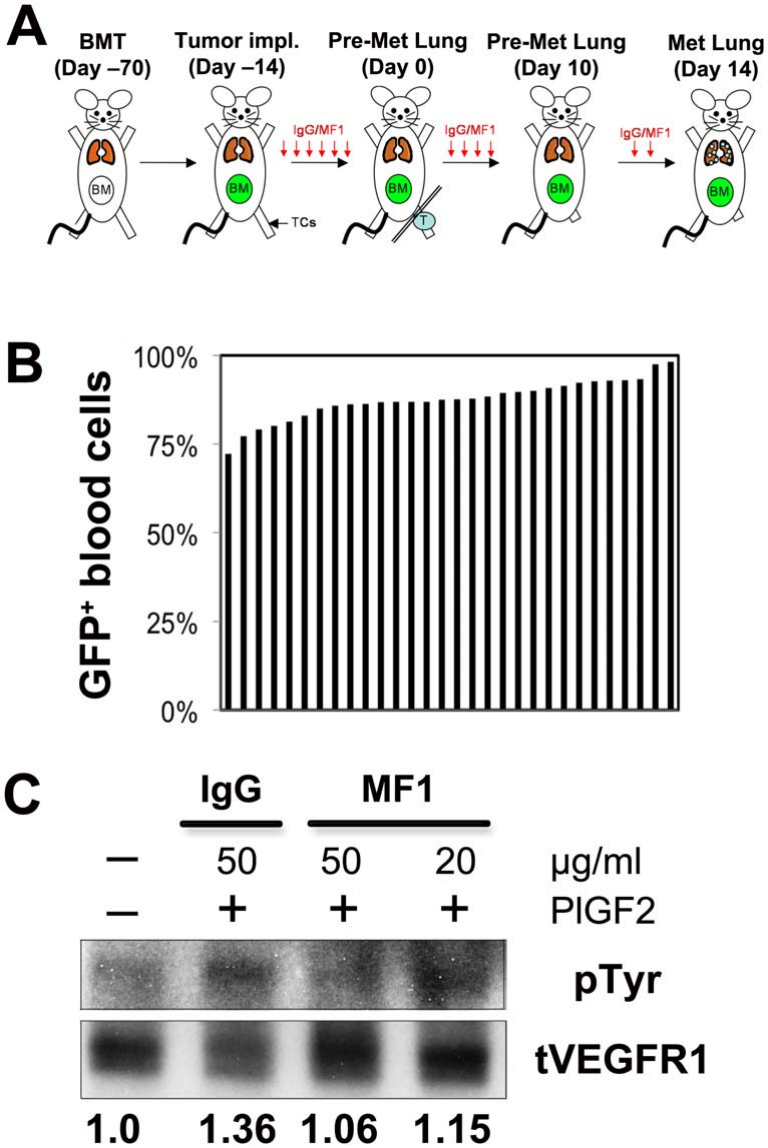
Experimental design. (A) Timeline for spontaneous metastasis studies: C57BL mice were lethally irradiated and transplanted 24 hours later with bone marrow cells isolated from *Actb-GFP*/C57BL mice (BMT). BMT mice were allowed to recover for 8 weeks prior to study. LLC1 or B16 tumor cells (TCs) were subcutaneously (s.c.) implanted in the left leg. Three days after LLC1 or B16 injection and until sacrifice, mice were treated 3 times per week by intraperitoneal (i.p.) injection of rat IgG (control) or VEGFR1-blocking antibody (MF1, 20 mg/kg). Primary tumors were resected when they reached a maximum length of 10 mm. Lung tissue was isolated from mice at three time points: at time of primary tumor resection and 10 and 14 days after removal of the primary tumor. Note: Tumor growth varied from 13–17 days; no macroscopic metastases are detectable at days 0 or 10 after tumor resection. (B) Bone marrow chimerism (each blue line represents GFP^+^ BMDCs in one BMT mouse, 30 mice prepared in one BMT procedure). Blood flow cytometric analysis was used to enumerate the percentage of GFP^+^ BMDCs among nucleated blood cells. (C) Mouse brain endothelial cells treated with rat IgG or MF1 were stimulated with 100 ng/ml of recombinant mouse PlGF-2 and incubated with anti-VEGFR1 antibody (MF1). VEGFR1 was immunoprecipitated and the ratio of phosphorylated-VEGFR1 to total VEGFR1 was compared to the ratio calculated for non-stimulated endothelial cells (assigned the value of 1). MF1 inhibited VEGFR1 phosphorylation in endothelial cells after PlGF2 stimulation in a dose-dependent manner.

C57BL mice (6–7 weeks old, male) were lethally irradiated (^137^Cs Irradiator; Atomic Energy of Canada Ltd, Mississauga, Canada) using two 6 Gy fractions (with less than 12 hour time interval between dosing) delivered to the whole body. Irradiated mice were rescued 24 hours later by a bone marrow transplant isolated from *Actb-GFP*/C57BL, as previously described [13] (**Figure 1A**). We used the model after confirming that no weight loss or fibrosis, inflammation or any other sign of damage was detectable in the lungs two months after BMT. The BMT protocol was also optimized to ensure reproducible levels of GFP^+^ BMDC chimerism in BMT-*Actb-GFP*/C57BL mice. Blood was collected at four and eight weeks after BMT for flow cytometry analysis. To limit the variability in GFP-BMDC chimerism, BMT mice were prepared in large groups (32 recipient mice were injected with pooled BM from 11 donor mice). One group was prepared for each tumor model (see **Figure 1B**).

### Tumor Cells

Lewis lung carcinoma (LLC1/LL2, CRL-1642) and B16 melanoma cell lines (CRL-6323) – both syngeneic to C57BL mouse – were purchased from ATCC (Manassas, VA). Both cell lines were propagated in DMEM (LLC1-ATCC/B16F1-Cellgro) supplemented with 10% fetal bovine serum (Atlanta Biologicals, Norcross, GA).

### Tumor Growth and Metastasis Model

LLC1 or B16 cells were subcutaneously (s.c.) implanted in the left leg of BMT-*Actb-GFP*/C57BL (8 weeks after BMT), *flt-1* ^TK–/–^/C57BL or C57BL mice as a 50 µl concentrated cell solution containing 300,000 cells in sterile PBS. Tumor growth was measured with a caliper three times per week and tumor volume (V) was calculated using the following formula (**1**):
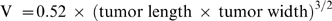
(1)


**Figure 2:**
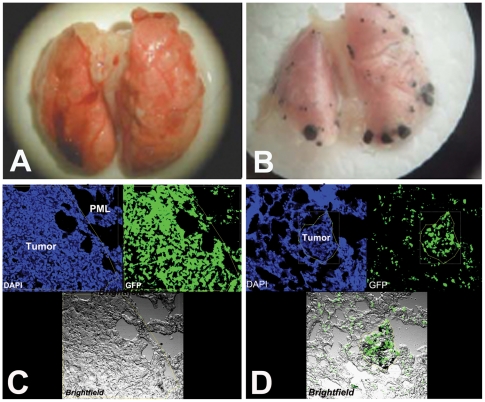
Effect of VEGFR1-blockade on metastasis. Representative images of lung metastases 14–16 days after resection of the primary tumor and bone marrow-derived cell (BMDC) accumulation in the primary tumor, peri-metastatic lung (PML, tissue surrounding lung metastases) and lung metastases in the LLC1 (A) and B16 (B) models. The number of BMDCs was calculated as the ratio of green fluorescence protein (GFP)-surface area to DAPI-surface area. DAPI was used to stain the nuclei of all cells (n = 6–8 mice per group). All images are 512 µm across.

Primary tumors were surgically resected when they reached a diameter of 10 mm. Mice were sacrificed by pentobarbital overdose (200 mg/kg administered by i.p. injection) at the time of resection (i.e., day 0), or 10 or 14 days after resection to analyze BMDC infiltration and metastatic nodule formation in the lungs (see schema in **Figure 1A**). Prior to sacrifice, blood was collected from anesthetized animals by cardiac puncture using a 26-G needle. The lungs were washed by cardiac injection of 15 ml of PBS, weighed and then examined using a dissecting microscope. The number of macroscopic metastases was enumerated by counting individual nodules with a cell counter. B16 metastases are easily identifiable, in part due to their pigmentation (see **Figure 2**). Primary tumor tissues and whole lungs were fixed in 4% paraformaldehyde at 4°C for 6 hours, dehydrated in 30% sucrose overnight, and embedded in OCT into frozen samples for immunofluorescence histological analyses.

### Antibody Treatment

Tumor-bearing mice were administered i.p. MF1 blocking monoclonal antibodies against VEGFR1 (a gift from ImClone Systems, New York, NY) at a blocking dose of 20 or 40 mg/kg three times per week (see Ref. [20]) from the time of tumor implantation (**Figure 1B**). As a control, tumor-bearing mice were treated i.p. 3 times per week with 20 mg/kg of non-specific rat IgG (Jackson ImmounoResearch Laboratories, West Grove, PA). To confirm MF1 inhibitory activity, we incubated mouse brain endothelial cells (bEnd.3 cells) in serum free condition overnight. After 1 hr of exposure with rat IgG (50 µg/ml) or MF1 (20 µg/ml or 50 µg/ml), cells were stimulated with 100 ng/ml recombinant mouse PlGF-2 (Minneapolis, MN) for 10 min. Then, the cells were rinsed with cold PBS and collected using RIPA buffer supplemented with protease inhibitors (Roche, Nutley, NJ) and phosphatase inhibitiors cocktail (Sigma, St. Louis, MO). Protein extracts were incubated with anti-VEGFR1 antibody (Santa Cruz Biotechnology, Santa Cruz, CA) overnight and VEGFR1 was precipitated using protein A agarose. Immunoprecipitates were separated on denaturing gel and transferred to PVDF membrane. Phosphotyrosine was detected using anti-phosphotyrosine antibody (Millipore, Billerica, MA). The membrane was then stripped and re-probed with anti-VEGFR1 antibody (**Figure 1C**).

### Histological Analysis

Frozen tumor and lung tissue samples were cryosectioned into 10-20 µm slices. Tissue sections were immunostained with monoclonal antibodies against CD11b-PE, F4/80-PE, and CD31-APC (all from BD Pharmingen, San Diego, CA). For VEGFR1 staining, we used MF1 antibodies labeled with Alexa-Flour-647 (Molecular Probes, Carlsbad, CA). All tissue sections were mounted on glass coverslips using Vectashield® mounting media with DAPI from Vector Labs (Burlingame, CA). For quantitative analysis, we collected 6–12 random 8-bit images per section of lung (6 per region of the lung, including lung metastasis and tissue surrounding metastasis) and tumor tissue (6 images of the periphery and 6 images of the center) (1024×1024 pixels), using a confocal microscope (Olympus, Center Valley, PA) and a 20× water-immersion lens (0.95NA). For each image, we determined the area occupied by GFP-positive BMDCs or immunostained cells normalized by area of DAPI-stained cells (nuclear counterstaining) using an algorithm written for Matlab software. The mean ratio of GFP to DAPI coverage area was calculated as a measure of cell number per tissue area. We report the average of the mean values of GFP/DAPI ratio for mouse lungs or tumors in each treatment group (6–7 lungs or tumors per treatment group were included).

### Flow Cytometric Analyses

Blood was collected from mice by cardiac puncture. Tumor tissues were digested into a single cell suspension using collagenase type II (Worthington, Lakewood, NJ) [13]. Cells were immunostained with the following monoclonal antibodies: anti-CD11b-APC, anti-Gr-1-FITC/PE, anti-CXCR4-PE, anti-CD45-PerCP (all from BD Pharmingen) and MF1-Alexa-647. We used Fc-blocking antibodies (BD Pharmingen) to block non-specific binding and non-specific fluorescently labeled IgG as control.

### Statistical analysis

Two-tailed unpaired students t-tests (assuming unequal variances) were used to compare all treatment and control groups with p<0.05 indicating a statistical difference. The rate of spontaneous metastasis formation in control and treatment groups was also compared using the Mann-Whitney U test (Wilcoxon rank-sum test).

## Results

### Effect of VEGFR1 blockade on primary tumor growth

Continuous blockade of VEGFR1 with MF1 antibodies – from the time of LLC1 or B16 implantation in BMT-*Actb-GFP*/C57BL mice – did not delay primary tumor growth compared to non-specific IgG (**Figure S1A,B**). Similarly, LLC1 tumor growth rate was comparable in *flt-1* ^TK–/–^/C57BL to that in C57BL mice (**Figure S1C**). The growth of B16 melanoma was delayed by an average of 2 days in *flt-1* ^TK–/–^/C57BL compared to C57BL mice (p<0.05, **Figure S1D**). Thus, blockade of VEGFR1 activity leads to a slight or no growth delay in primary B16 and LLC1 tumors. Since VEGFR1 is thought to modulate BMDCs recruitment to tumors and metastasis, we measured next the number of metastases that formed in the lungs after surgical removal of the primary tumors.

### Effect of VEGFR1 blockade on spontaneous metastasis formation after primary tumor resection

We surgically removed the primary tumors – as per animal protocol – when LLC1 and B16 tumors grew to 10-mm in diameter (approximately 15–17 days after implantation). Two weeks after resection of primary tumors, metastatic tumor nodules were present in the lungs of all 17 mice bearing LLC1 tumors, both after MF1 (n = 9) and IgG (n = 8) treatment (see data reported in Ref. [17]). The metastatic nodules (with sizes estimated as 1–3 mm, 3–5 mm, >5 mm) were counted using a dissecting microscope (**Figure 2**). At this time-point, there were no significant differences between the number nor the size of metastatic nodules [17]. At the same time-point after resection of primary B16 tumors, 9/12 mice treated with MF1 and 9/13 mice treated with IgG developed lung metastatic nodules. In mice that developed macroscopic metastases, there was no significant difference between the numbers of nodules in the MF1-treated and IgG-treated groups [17]. Thus, in this setting of neoadjuvant and adjuvant VEGFR1 blockade (i.e., continuous blockade of VEGFR1 using MF1 from the time of primary tumor implantation) did not significantly alter the rate of spontaneous macroscopic lung metastasis formation.

Next, we measured metastatic nodule formation after resection of LLC1 or B16 primary tumors implanted in *flt-1* ^TK–/–^/C57BL mice (mice which lack the tyrosine kinase domain but not the extracellular domain [18]) or in C57BL mice. When evaluated at 14 days after primary LLC1 tumor resection, 12/13 *flt-1* ^TK–/–^/C57BL mice and 6/8 C57BL mice had macroscopic lung metastases. At the same time-point after B16 primary tumor resection, 7/14 *flt-1* ^TK–/–^/C57BL mice and 6/8 C57BL mice had metastatic nodules in the lung (see data reported in Ref. [17]). Control and treatment groups were compared using student's t-tests and rank-sum tests (Mann-Whitney U), and differences in metastatic tumor formation were not significant between groups. Thus, genetic ablation of VEGFR1 activity did not result in significantly different rate of formation of macroscopic lung metastasis. Similarly, the number of spontaneous macroscopic lung metastases formed 2 weeks after LLC1 or B16 resection was not significantly different between *flt-1* ^TK–/–^/C57BL and C57BL mice [17].

### The number of circulating VEGFR1^+^ and CXCR4^+^ BMDCs is tumor-dependent, but blockade of VEGFR1 activity does not change BMDC accumulation in the primary tumor

**Figure 3:**
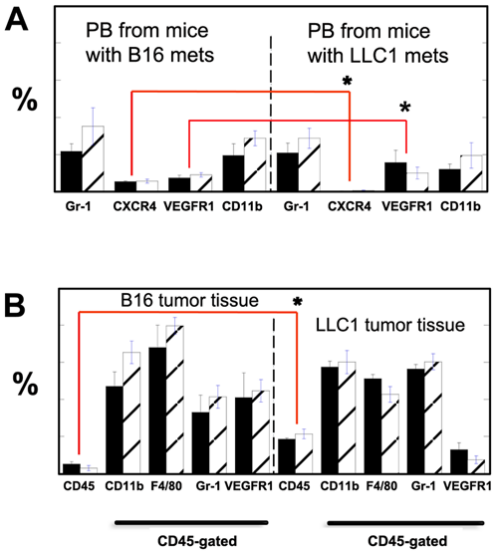
Flow cytometric analysis of circulating cells and tumor-homing BMDCs in tumor-bearing mice. (A) Peripheral blood from C57BL (black bars) and *flt-1* ^TK–/–^/C57BL (empty bars with diagonal lines) mice were analyzed using antibodies for specific surface markers (all cells were CD45^+^). B, Flow cytometric analysis of enzymatically digested LLC1 tumor suspensions from C57BL (black bars, n = 6) and *flt-1* ^TK–/–^/C57BL (empty bars with diagonal lines, n = 6) mice using antibodies for specific surface markers. There was no statistically significant difference in the blood cell population studied in C57BL and *flt-1* ^TK–/–^/C57BL nor in the number of hematopoietic cells within the tumor tissues. However, there were significant inter-tumor differences: mice with B16 tumors had significantly more CXCR4^+^CD45^+^ blood circulating cells than mice with LLC1 tumors (p<0.05 by Student's t-test). In addition, while B16 tumors recruited fewer CD45^+^ cells, the fraction of VEGFR1^+^CD45^+^ cells was greater than in LLC1 tumors. A statistically significant difference (p<0.05 by Student's t-test) is identified with an asterisk.

Several studies have shown that VEGFR1 modulates BMDC infiltration in tumors, and that BMDCs are modulating metastasis. Thus, we measured the accumulation of BMDCs in the primary tumors and lungs after the formation of macroscopic metastases in our model. Evaluation of blood cells in tumor-bearing *flt-1* ^TK–/–^/C57BL and C57BL mice showed no significant difference between circulating CD45^+^, CD11b^+^, Gr-1^+^, VEGFR1^+^ or CXCR4^+^ cells in these mice (**Figure 3A**). However, we detected significantly more circulating VEGFR1^+^ cells and significantly fewer circulating CXCR4^+^ cells in C57BL mice bearing LLC1 tumors compared to C57BL mice bearing B16 tumors. Next, we investigated the intra-tumor accumulation of BMDCs. While LLC1 or B16 cells do not express VEGFR1 (as evaluated by PCR, data not shown), certain cells in the tumor stroma might express VEGFR1 (e.g., endothelial cells). Thus, we performed flow cytometric analysis of tumor stromal cells in LLC1 and B16 tumors after gating on the CD45^+^ cell population. LLC1 tumors contained more hematopoietic (CD45^+^) cells than B16 tumor both in *flt-1* ^TK–/–^/C57BL and C57BL mice. However, no statistically significant differences were found among infiltrating hematopoietic BMDCs positive for VEGFR1^+^ cells, Gr-1 (granulocytes/monocytes), F4/80 (macrophages) or CD11b (all myeloid cells) in the tumors grown in *flt-1* ^TK–/–^/C57BL and C57BL mice (**Figure 3B**).

### VEGFR1-blocking antibody reduces the accumulation of bone marrow-derived cells in the metastatic lesions and peri-metastatic lung tissue, but not in the primary tumors

**Figure 4:**
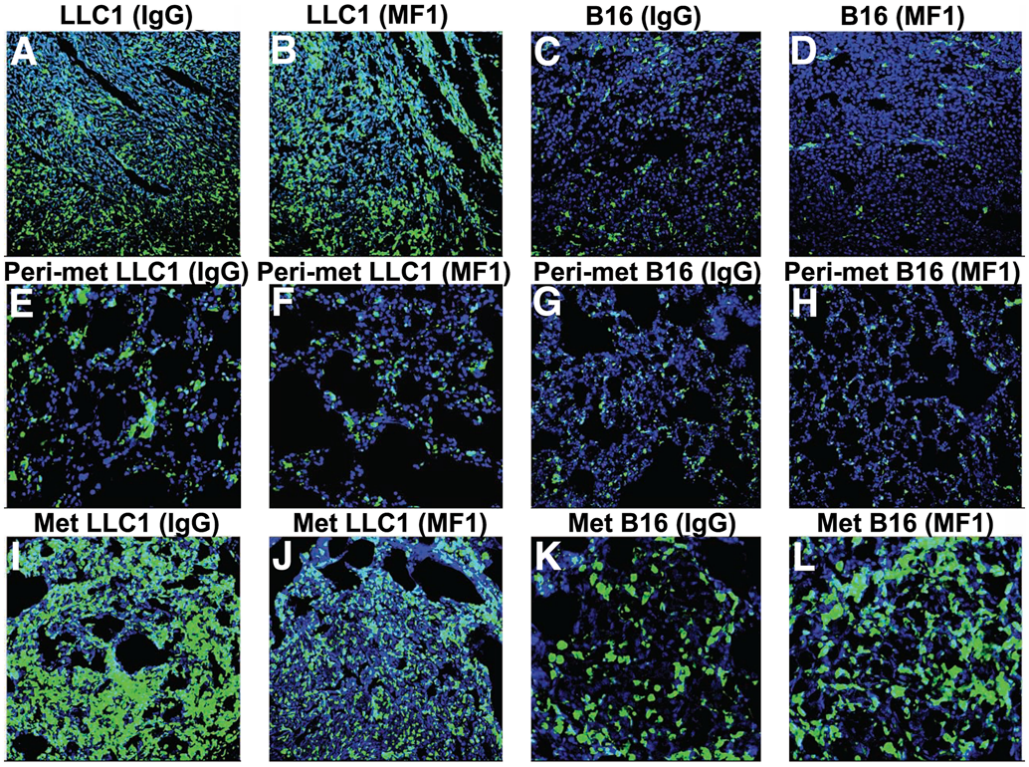
Bone marrow-derived cell (BMDC) accumulation in the primary tumors, lung metastases and surrounding (peri-metastatic) lung tissues after VEGFR1 blockade. Confocal images of cryo-sectioned primary tumor (A–D), peri-metastatic lung tissue (E–H) and metastatic lung tumors (I–L) collected from BMT-*Actb-GFP*/C57BL mice (BMDCs are shown in green) after IgG (A, C, E, G, I, K) or MF1 (B, D, F, H, J, L) treatment. Tissues were counterstained with DAPI nuclear dye (in blue). The width of images in A–J is 512 µm and images in K and L are 256 µm across.

To establish with precision the BMDCs infiltration after VEGFR1 blockade, we quantified the BMDCs in LLC1 and B16 tumors implanted in BMT-*Actb-GFP*/C57BL mice after treatment with MF1 at the time of resection (approximately 2 weeks after implantation). Antibody blockade of VEGFR1 – from the time of implantation – did not change the number of GFP^+^ BMDCs in primary LLC1 or B16 tumors (**Figure 4A–B**) [17]. However, when most mice spontaneously developed macroscopic metastases (2 weeks after primary tumor resection), we detected a significant increase in BMDC accumulation inside the LLC1 metastatic nodules and in the peri-tumor lung tissue, but not in B16 metastases (**Figures 2** and **4E,L**). Thus, the reduction by VEGFR1 blockade in BMDC accumulation in metastases is tumor dependent. However, prior to the time-point used by us for resection (days 14–16), BMDC “pre-metastatic niches” [10], and metastatic foci [13] should have been formed in the lungs in these tumor models. Thus, we measured the accumulation of BMDCs in the lungs at the earlier time points (i.e., days 0 and 10 after resection).

### VEGFR1-blocking antibody does not reduce the baseline accumulation of myeloid bone marrow-derived cells in the pre-metastatic lungs

**Figure 5:**
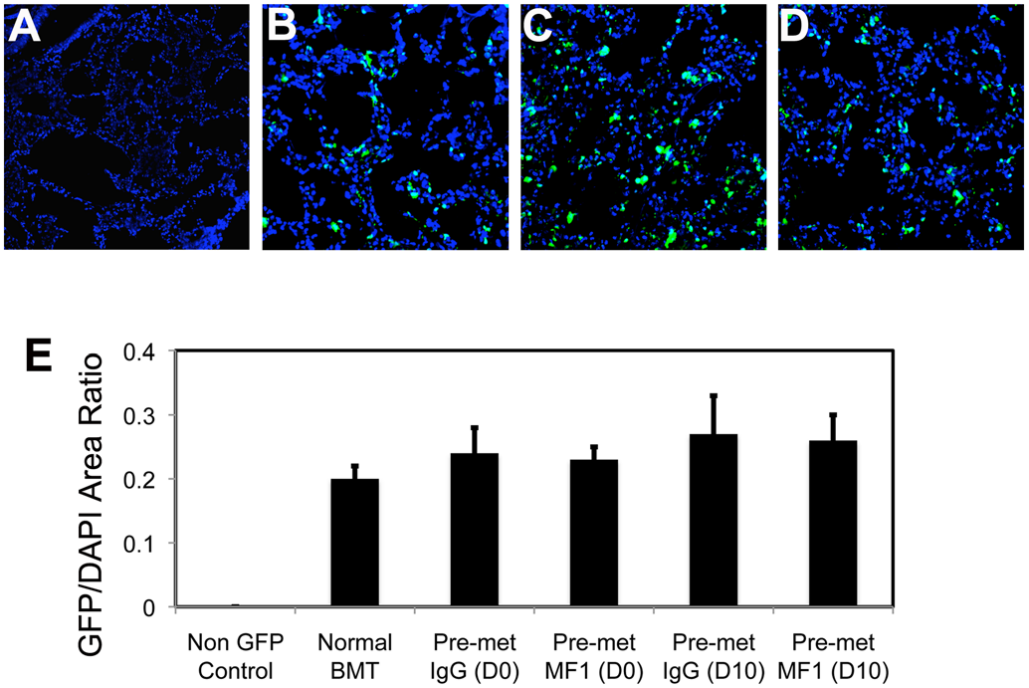
Effect of VEGFR1-blockade on BMDC infiltration in lungs prior to macroscopic metastasis formation. Treatment of tumor bearing mice with MF1 antibody did not change the infiltration with BMDCs in lungs at the time of primary tumor resection (day 0) or 10 days after primary tumor removal. Representative images of lung tissue from non-tumor bearing C57BL (A) and BMT-*Actb-GFP*/C57BL (B) mice and from LLC1-tumor bearing mice at the time of primary tumor resection (day 0) after treatment with IgG (C) or MF1 (D). The number of BMDCs was calculated as the ratio of green fluorescence protein (GFP)-surface area to DAPI-surface area (E). DAPI was used to stain the nuclei of all cells (n = 6–8 mice per group). All images are 512 µm across.

**Figure 6:**
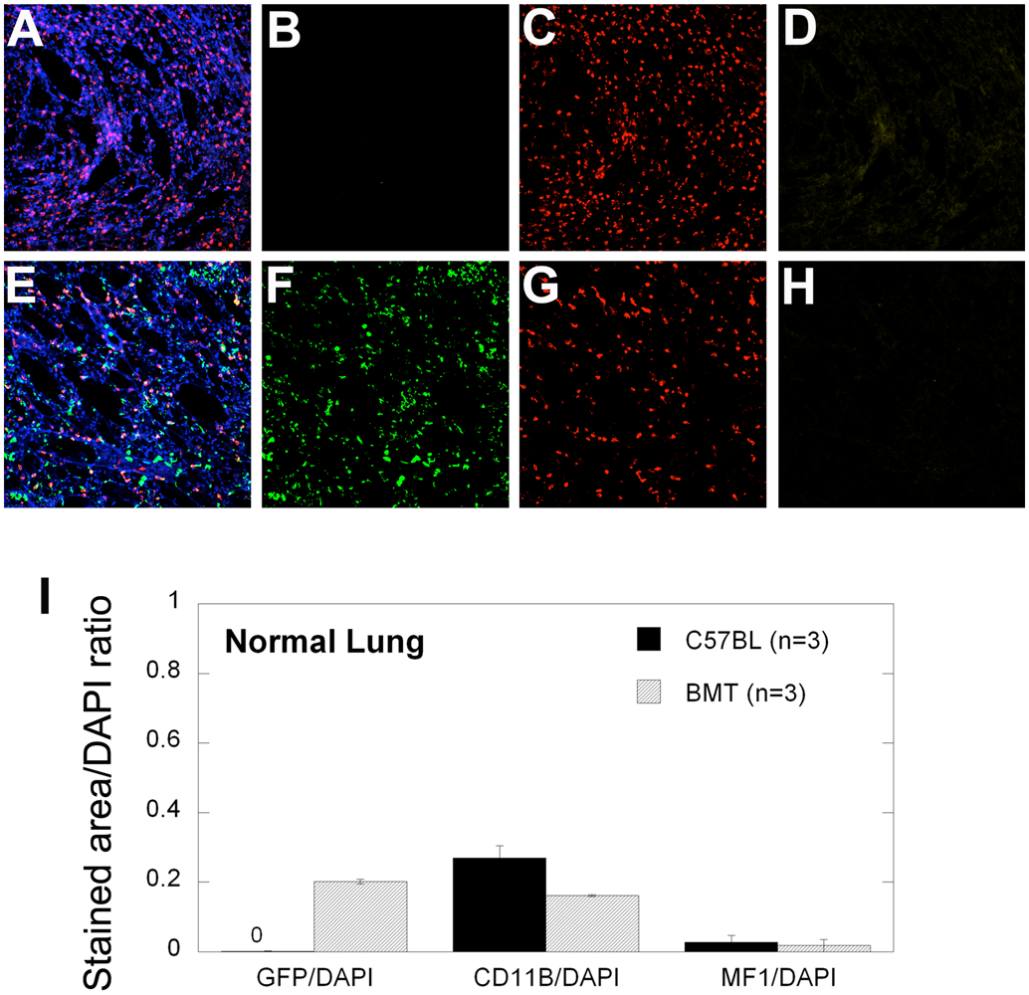
Representative images and analysis of CD11b and MF1 staining of normal lung tissue. (A–H) Confocal microscopy images of lung tissue from C57BL (A–D, non-irradiated, non-GFP control) and BMT-*Actb-GFP*/C57BL (E–H, irradiated mice, GFP^+^ BMDCs shown in green) mice stained with CD11b-AF546 (red) and MF1-AF647 (yellow) and counterstained with DAPI nuclear dye (blue). The number of GFP, CD11b, or MF1 positive cells was calculated as a ratio of green, red, or yellow surface area to DAPI surface area, respectively (I). All images are 512 µm across.

**Figure 7:**
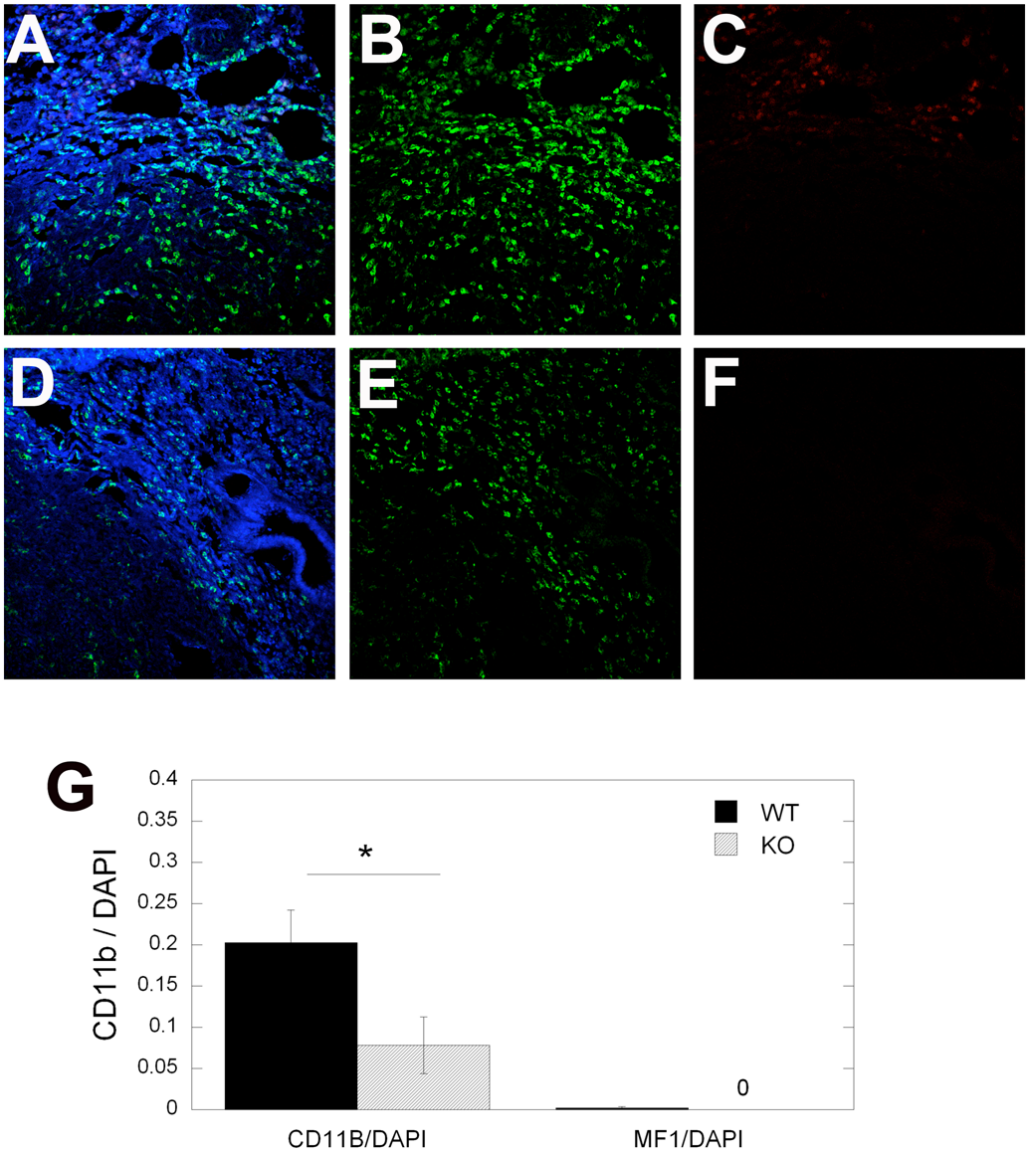
Representative images and quantification of CD11b and VEGFR1 positive cells at the periphery of lung metastases. (A–F) Confocal microscopy images of lung tissue from C57BL mice (WT, A–C) and *flt-1* ^TK–/–^/C57BL (KO, D–F) mice after immunostaining for CD11b (using FITC-labeled anti-CD11b antibody, in green in B and E), or for VEGFR1 (using AF647-labeled MF1 antibody, in red in C and F). Sections were counterstained with DAPI nuclear dye (in blue in A–F). The number of positive cells was calculated as the ratio of CD11b^+^ or VEGFR1^+^ surface area to DAPI surface area (G). A statistically significant difference (p<0.05 by Student's t-test) is identified with an asterisk. All images are 512 µm across.

At the time of the primary tumor resection, as well as 10 days after that, evaluation of lungs showed no macroscopic metastatic tumor nodules. Nevertheless, BMDC infiltration at the time of resection was small but detectable in both LLC1 and B16 tumor models, and comparable with BMDC accumulation in tumor-free BMT-*Actb-GFP*/C57BL mice (**Figure 5**). Thus, VEGFR1 blockade by MF1 treatment did not reduce BMDC infiltration in the lungs prior to macroscopic metastases formation (i.e., at days 0 and 10) in mice that had primary tumors removed [17]. These BMDCs were likely pulmonary alveolar macrophages, which reside in the normal, non-irradiated lung in comparable numbers in tumor-free non-BMT C57BL mice (**Figure 6**). To directly address the issue of BMDC phenotype (**Figure 7A,D**), and to exclude the possibility that inflammatory BMDCs infiltration in lungs was an artifact due to prior whole body irradiation and BMT, we performed CD11b (Mac1) and VEGFR1 immunostaining in normal, non-irradiated C57BL and *flt-1* ^TK–/–^/C57BL mice. In the lungs of these mice, we detected myeloid (CD11b^+^) cells in numbers comparable to those of lung infiltrating BMDCs in BMT-*Actb-GFP*/C57BL mice. Moreover, the number of VEGFR1^+^ cells in the lung tissue was not significantly different between C57BL and *flt-1* ^TK–/–^/C57BL mice (**Figure 7B,E**). Next, we measured the number of CD11b^+^ cells in spontaneous metastatic nodules in *flt-1* ^TK–/–^/C57BL and C57BL mice formed after primary tumor resection. Consistent with the effect of antibody blockade of VEGFR1, we detected a significant decrease in the number of CD11b^+^ cells but not VEGFR1^+^ cells in the peri-tumor areas in LLC1 lung metastases from (non-irradiated) *flt-1* ^TK–/–^/C57BL and C57BL mice (**Figure 7C,F,G**).

## Discussion

VEGF is a clinically validated target in metastatic (advanced) cancer treatment, but the mechanism(s) by which its blockade leads to a clinical benefit remain unclear [3]. It is largely thought that the benefit derives from blockade of the interaction between VEGF and its receptor VEGFR2 on endothelial cells, which mediates key functions in angiogenesis and vascular function [2]. But VEGF binds with a higher affinity to VEGFR1, which is present on endothelial cells but also on inflammatory cells in tumors or even on the malignant cells in certain tumors [1]. Recent studies of the VEGFR1 ligand PlGF have proposed that VEGFR1 mediates tumor growth and angiogenesis by recruiting tumor-promoting inflammatory cells [7]. Others have shown that VEGFR1 signaling activation leads to MMP-9 expression in lung stromal cells, which facilitates lung metastasis in a model of experimental metastasis [19]. Moreover, it has been proposed that BMDC infiltration into lung tissue precedes the spontaneous arrival of metastatic cancer cells, and that VEGFR1 expression on the BMDCs mediates this process [10]. The direct implication is that VEGFR1 blockade may prevent/eradicate metastasis of the primary tumors to distant organs. Of note, a phase III of the anti-VEGF antibody bevacizumab as adjuvant after surgery in patients with colorectal cancer showed that VEGF blockade did not affect metastasis formation or patients' disease-free survival [21].

We sought to establish if blockade of VEGFR1 activity could eradicate tumor progression to metastasis in a model mirroring the neoadjuvant and adjuvant therapy of tumors (i.e., continuous VEGFR1 blockade in mice from the time of implantation of the primary tumor, including the period after resection of the primary tumor when it has reached 1 cm in diameter and has seeded metastatic cells in the lungs). None of the cancer cell lines used in this study (LLC1 and B16) expressed detectable levels of VEGFR1. In these models, we measured the spontaneous formation of lung metastatic nodules. In addition, we studied formation of lung metastatic nodules in a genetic model of VEGFR1 deficiency (*flt-1* ^TK–/–^/C57BL mice, which lack tyrosine kinase domain of VEGFR1). Since these mice express the extracellular domain of VEGFR1, the ligands can bind to the extracellular portion of the receptor, unlike when the MF1 antibodies are used (which completely block both ligand-receptor interaction and downstream signaling). Consistent with published reports on blockade of the VEGFR1 ligand PlGF, we found a significantly decreased blood vessel density in primary LLC1 tumor after VEGFR1 blockade by MF1 treatment. However, MF1 treatment did not decrease the number of BMDCs recruited into primary LLC1 tumors, nor their growth rate. Moreover, despite continued VEGFR1 blockade with MF1, metastatic nodule formation rate was not affected in mice whose tumors were resected when they reached 1 cm in diameter. These results were confirmed using the spontaneous LLC1 metastasis in *flt-1* ^TK–/–^/C57BL mice. In a second model, we evaluated B16 growth and spontaneous metastasis after primary tumor resection. Similar to LLC1 tumors, tumor growth and spontaneous B16 metastatic nodule formation was not significantly affected by MF1 treatment from the time of tumor implantation. In *flt-1* ^TK–/–^/C57BL mice, B16 primary tumor growth was significantly delayed compared to C57BL, but spontaneous B16 metastatic nodule formation after primary tumor resection was similar. The lack of effects of VEGFR1 antagonism on the rate of spontaneous metastasis is of particular interest in light of recent reports that VEGF antagonism might lead to increased metastatic burden [22], [23].

In a study of PlGF blockade, Fischer *et al*. reported significant effects of anti-PlGF on the primary B16 tumors and lymphatic metastasis [7]. Data from our own experiments using PlGF blockade (Dawson et al., unpublished observations) are consistent with the results in this report [7], and suggest that the antiangiogenic effect in primary tumors may account for the effects of PlGF blockade on lymphatic metastasis and lung metastasis. It is important to note that in this case the blockade of the ligand (i.e., PlGF) may affect not only VEGFR1 activity, but also NRP1 and NRP2 [1].

We used the similar regimen for MF1 treatment, similar cell lines and similar mouse strain as Kaplan et al. [10]. At this dose, MF1 was “biologically active” since it significantly inhibited PlGF-induced VEGFR1 phosphorylation *in vitro*. Moreover, MF1 treatment significantly inhibited BMDC accumulation in and around growing metastatic nodules *in vivo*. Nevertheless, VEGFR1 activity blockade did not change BMDC/CD11b^+^ cell accumulation in pre-metastatic lungs. This may point toward a key role of the resident pulmonary alveolar macrophages in the pre-metastatic phase as opposed to *de novo* BMDC recruitment. The lack of effect of MF1 treatment on tumor angiogenesis and inflammatory cell infiltration has been well established for spontaneous tumors (e.g., pancreatic insulinoma, see Ref. [8]). In models in which an anti-tumor effect for VEGFR1 blockade was detected, they were attributed to direct effects on cancer cells or by modulation of angiogenesis [20], [24], but there was no data reported on hematogenous metastasis formation. This may be related to cell migration and MMP-9 activity in response to VEGFR1 activation in resident pulmonary macrophages and/or endothelial cells [19].

The regulation of tumor angiogenesis by VEGFR1 may be direct or indirect (related to BMDC recruitment) [1], [4]. Given the lack of modulation of metastatic nodule formation by VEGFR1 in our models, we evaluated the kinetics of BMDC infiltration in lungs prior to and after macroscopic metastatic nodule formation. We found no significant difference after blockade of VEGFR1 activity in BMDC infiltration in lungs prior to macroscopic metastasis formation. BMDC infiltration in BMT-*Actb-GFP*/C57BL mice was similar the CD11b^+^ cell infiltration in non-irradiated C57BL mice. Moreover, MF1 treatment did not significantly change the number of BMDCs in the pre-metastatic lungs of mice. This lack of modulation of BMDC infiltration in normal lungs was confirmed in *flt-1* ^TK–/–^/C57BL mice, which had comparable CD11b^+^ cell numbers (most likely pulmonary alveolar macrophages) in pre-metastatic lungs. Nevertheless, after the onset of metastatic nodule growth, MF1 blockade of VEGFR1 led to a partial decrease in BMDC infiltration inside and around LLC1 metastatic nodules, which is consistent with modulation by VEGFR1 activity of BMDC accumulation in some tumors during their growth. Of note, despite the significant reduction, the BMDC accumulation was not completely blocked, and remained quite high, suggesting that BMDC accumulation in growing metastatic nodules is only partially controlled by VEGFR1 signaling. In growing B16 tumors, which have low levels of BMDC infiltration in both primary and metastatic sites [17], MF1 blockade of VEGFR1 did not change the number of BMDCs. Collectively, these data suggest that signaling pathways alternative to VEGFR1 are involved in BMDC infiltration in growing B16 or LLC1 tumors. Of interest, in BMT-*Actb-GFP*/C57BL mice, GFP^+^ expression in BMDCs often co-localized with expression of Gr1 or CD11b. These BMDCs have been shown to modulate resistance to VEGF blockade in these tumor models [25].

In summary, we show that formation of the metastatic nodules is independent of intracellular VEGFR1 activity, since neither pharmacologic blockade nor genetic deficiency in intracellular VEGFR1-TK domain prevented or altered pre-metastatic BMDC infiltration, nor spontaneous metastasis. We propose that pathways other than VEGFR1 are activated and lead to BMDC infiltration, and should be targeted in order to optimize anti-VEGF therapy and prevent spontaneous metastasis.

## Supporting Information

Figure S1 Primary LLC1 and B16 tumor growth kinetics after VEGFR1 blockade. LLC1 and B16 tumors were grown in BMT-Actb-GFP/C57BL mice treated with IgG (black solid lines) or MF1 (blue dashed lines) (A,C) or in C57BL (WT, black solid lines) or flt-1TK-/-/C57BL (KO, blue dashed lines) mice (B,D).
(1.19 MB PDF)

## References

[1] Fischer C, Mazzone M, Jonckx B, Carmeliet P (2008). FLT1 and its ligands VEGFB and PlGF: drug targets for anti-angiogenic therapy?. Nat Rev Cancer.

[2] Ferrara N, Hillan KJ, Gerber HP, Novotny W (2004). Discovery and development of bevacizumab, an anti-VEGF antibody for treating cancer.. Nat Rev Drug Discov.

[3] Jain RK, Duda DG, Clark JW, Loeffler JS (2006). Lessons from phase III clinical trials on anti-VEGF therapy for cancer.. Nat Clin Pract Oncol.

[4] Rafii S, Lyden D, Benezra R, Hattori K, Heissig B (2002). Vascular and haematopoietic stem cells: novel targets for anti-angiogenesis therapy?. Nat Rev Cancer.

[5] de Visser KE, Eichten A, Coussens LM (2006). Paradoxical roles of the immune system during cancer development.. Nat Rev Cancer.

[6] Pollard JW (2004). Tumour-educated macrophages promote tumour progression and metastasis.. Nat Rev Cancer.

[7] Fischer C, Jonckx B, Mazzone M, Zacchigna S, Loges S (2007). Anti-PlGF inhibits growth of VEGF(R)-inhibitor-resistant tumors without affecting healthy vessels.. Cell.

[8] Casanovas O, Hicklin DJ, Bergers G, Hanahan D (2005). Drug resistance by evasion of antiangiogenic targeting of VEGF signaling in late-stage pancreatic islet tumors.. Cancer Cell.

[9] Kamoun WS, Ley CD, Farrar CT, Duyverman AM, Lahdenranta J Edema control by cediranib, a VEGF targeted kinase inhibitor, prolongs survival despite persistent brain tumor growth in mice.. J Clin Oncol.

[10] Kaplan RN, Riba RD, Zacharoulis S, Bramley AH, Vincent L (2005). VEGFR1-positive haematopoietic bone marrow progenitors initiate the pre-metastatic niche.. Nature.

[11] Erler JT, Bennewith KL, Cox TR, Lang G, Bird D (2009). Hypoxia-induced lysyl oxidase is a critical mediator of bone marrow cell recruitment to form the premetastatic niche.. Cancer Cell.

[12] Gerber HP, Condorelli F, Park J, Ferrara N (1997). Differential transcriptional regulation of the two vascular endothelial growth factor receptor genes. Flt-1, but not Flk-1/KDR, is up-regulated by hypoxia.. J Biol Chem.

[13] Duda DG, Cohen KS, Kozin SV, Perentes JY, Fukumura D (2006). Evidence for incorporation of bone marrow-derived endothelial cells into perfused blood vessels in tumors.. Blood.

[14] Gao D, Nolan DJ, Mellick AS, Bambino K, McDonnell K (2008). Endothelial progenitor cells control the angiogenic switch in mouse lung metastasis.. Science.

[15] Ohtaki T, Shintani Y, Honda S, Matsumoto H, Hori A (2001). Metastasis suppressor gene KiSS-1 encodes peptide ligand of a G-protein-coupled receptor.. Nature.

[16] Padua D, Zhang XH, Wang Q, Nadal C, Gerald WL (2008). TGFbeta primes breast tumors for lung metastasis seeding through angiopoietin-like 4.. Cell.

[17] Dawson MR, Duda DG, Fukumura D, Jain RK (2009). VEGFR1 activity-independent metastasis formation.. Nature: doi:10.1038/nature08254.

[18] Hiratsuka S, Minowa O, Kuno J, Noda T, Shibuya M (1998). Flt-1 lacking the tyrosine kinase domain is sufficient for normal development and angiogenesis in mice.. Proc Natl Acad Sci U S A.

[19] Hiratsuka S, Nakamura K, Iwai S, Murakami M, Itoh T (2002). MMP9 induction by vascular endothelial growth factor receptor-1 is involved in lung-specific metastasis.. Cancer Cell.

[20] Wu Y, Zhong Z, Huber J, Bassi R, Finnerty B (2006). Anti-vascular endothelial growth factor receptor-1 antagonist antibody as a therapeutic agent for cancer.. Clin Cancer Res.

[21] Wolmark N, Yothers G, O'Connell MG, Sharif S, Atkins JN (2009). A phase III trial comparing mFOLFOX6 to mFOLFOX6 plus bevacizumab in stage II or III carcinoma of the colon: Results of NSABP Protocol C-08.. J Clin Oncol.

[22] Ebos JM, Lee CR, Cruz-Munoz W, Bjarnason GA, Christensen JG (2009). Accelerated metastasis after short-term treatment with a potent inhibitor of tumor angiogenesis.. Cancer Cell.

[23] Paez-Ribes M, Allen E, Hudock J, Takeda T, Okuyama H (2009). Antiangiogenic therapy elicits malignant progression of tumors to increased local invasion and distant metastasis.. Cancer Cell.

[24] Luttun A, Tjwa M, Moons L, Wu Y, Angelillo-Scherrer A (2002). Revascularization of ischemic tissues by PlGF treatment, and inhibition of tumor angiogenesis, arthritis and atherosclerosis by anti-Flt1.. Nat Med.

[25] Shojaei F, Wu X, Malik AK, Zhong C, Baldwin ME (2007). Tumor refractoriness to anti-VEGF treatment is mediated by CD11b+Gr1+ myeloid cells.. Nat Biotechnol.

