# Supplementary material for: VEGFR1 Activity Modulates Myeloid Cell Infiltration in Growing Lung Metastases but Is Not Required for Spontaneous Metastasis Formation
Source: PLoS One. 2009 Sep 18;4(9):e6525. doi: 10.1371/journal.pone.0006525 (PMC2744279; doi:10.1371/journal.pone.0006525)
Supplement: Figure S1 — Primary LLC1 and B16 tumor growth kinetics after VEGFR1 blockade. LLC1 and B16 tumors were grown in BMT-Actb-GFP/C57BL mice treated with IgG (black solid lines) or MF1 (blue dashed lines) (A,C) or in C57BL (WT, black solid lines) or flt-1TK-/-/C57BL (KO, blue dashed lines) mice (B,D). (1.19 MB PDF) [file pone.0006525.s001.pdf]

Supplementary Figure Legend

Figure S1: Primary LLC1 and B16 tumor growth kinetics after VEGFR1 blockade. LLC1 and B16 tumors were grown in BMT-*Actb-GFP*/C57BL mice treated with IgG (black solid lines) or MF1 (blue dashed lines) (**A**,**C**) or in C57BL (WT, black solid lines) or *flt-1*$^{TK-/-}$/C57BL (KO, blue dashed lines) mice (**B**,**D**).

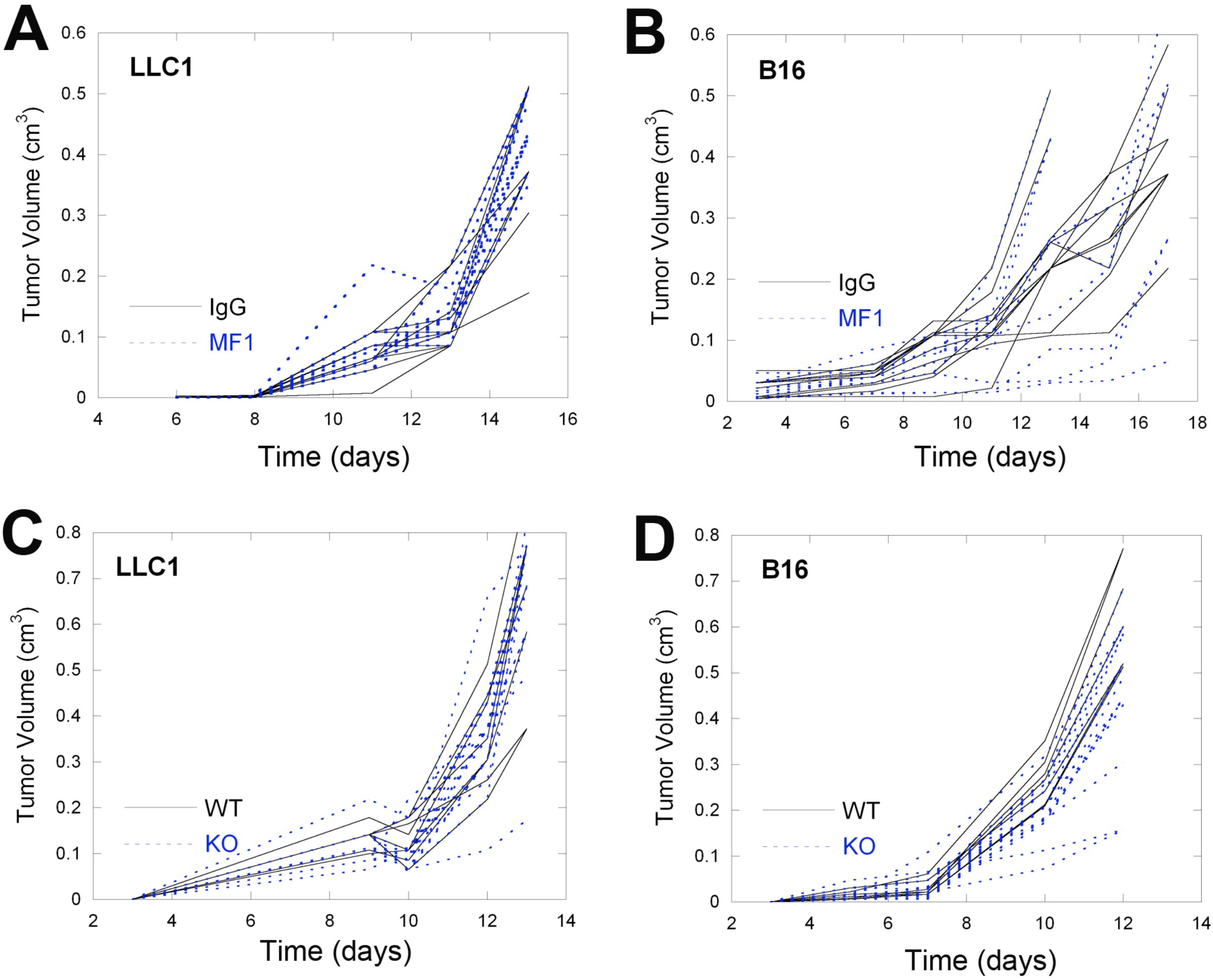
A
LLC1
Tumor Volume (cm3)
Time (days)
IgG
MF1
B
B16
Tumor Volume (cm3)
Time (days)
IgG
MF1
C
LLC1
Tumor Volume (cm3)
Time (days)
WT
KO
D
B16
Tumor Volume (cm3)
Time (days)
WT
KO
